# Supplementary material for: Testing the reliability of AI-generated protein structures
Source: bioRxiv. 2026 Jun 13:2026.06.11.731682. Preprint. [Version 1] doi: 10.64898/2026.06.11.731682 (PMC13278076; doi:10.64898/2026.06.11.731682)

bioRxiv preprint doi: https://doi.org/10.64898/2026.06.11.731682; this version posted June 13, 2026. The copyright holder for this preprint (which was not certified by peer review) is the author/funder, who has granted bioRxiv a license to display the preprint in perpetuity. It is made available under a CC-BY 4.0 International license.

Supplementary figure captions

Supplementary Figure S1. Genomic locations of the opposite-strand open reading frames (osORFs) for the eight osORFs with folding scores of 70 or above as reported by ColabFold. The genes containing the osORFs are, top to bottom and left to right: NKAIN3, FRK, OR14J1, SRRD, KHNYN, ORC4, OR2I1P, and ZNF354C. The exon-intron structure of the gene annotated in MANE is shown in blue across the top, with an alternative isoform with a shorter UTR, taken from the CHESS database, just below it. For OR14J1, OR2I1P, and ZNF354C, the CHESS annotation has the same UTR and is not shown. The position of the osORF is shown as a red rectangle for each gene.

Supplementary Figure S2. Protein structures predicted by ColabFold for the 18 osORFs in human protein-coding genes with pLDDT scores > 70, as visualized using PyMOL. Letters next to each structure correspond to the osORFs listed in Table 1.

bioRxiv preprint doi: https://doi.org/10.64898/2026.06.11.731682; this version posted June 13, 2026. The copyright holder for this preprint (which was not certified by peer review) is the author/funder, who has granted bioRxiv a license to display the preprint in perpetuity. It is made available under a CC-BY 4.0 International license.

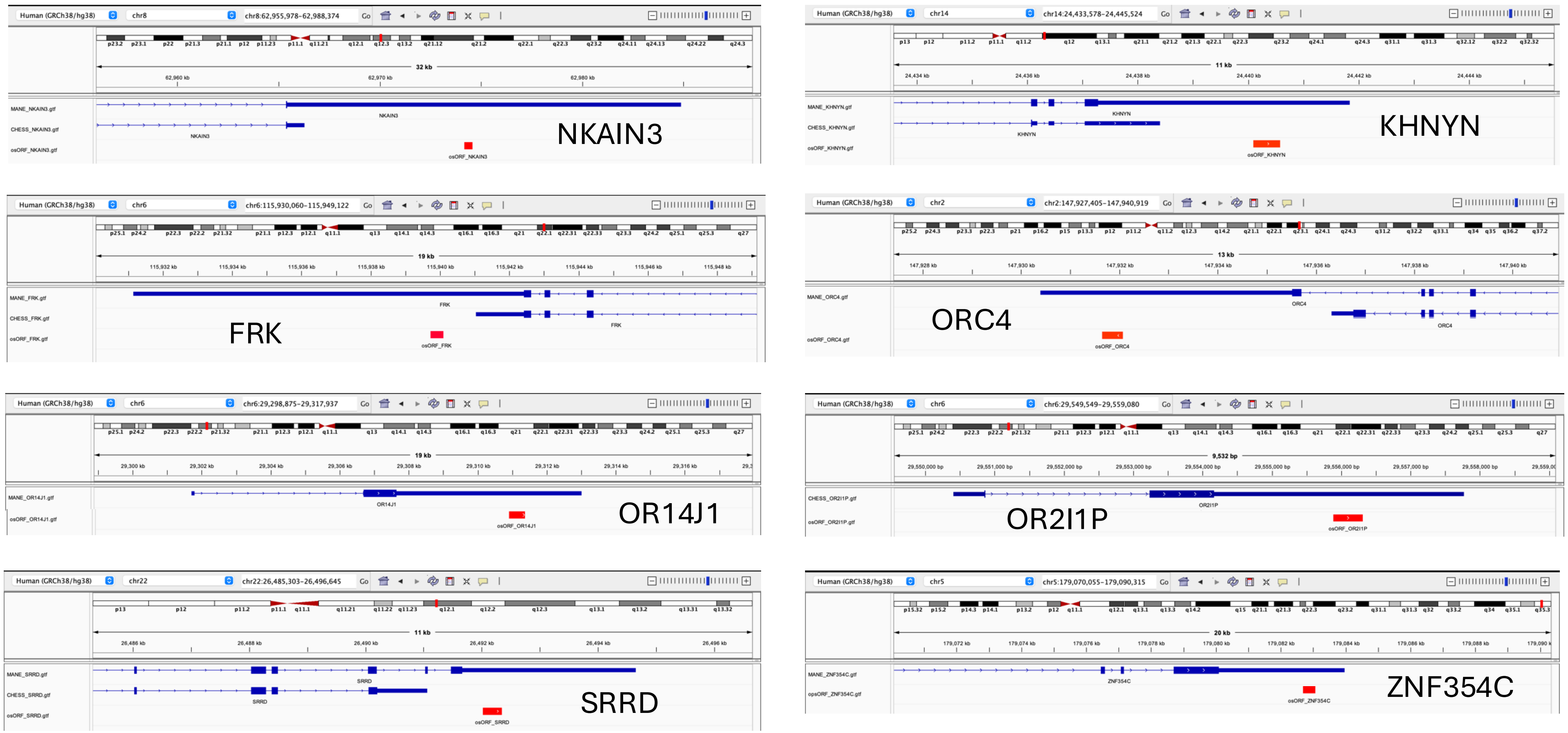
NKAIN3
KHNYN
FRK
ORC4
OR14J1
OR2I1P
SRRD
ZNF354C

bioRxiv preprint doi: https://doi.org/10.64898/2026.06.11.731682; this version posted June 13, 2026. The copyright holder for this preprint
CC-BY 4.0 International license

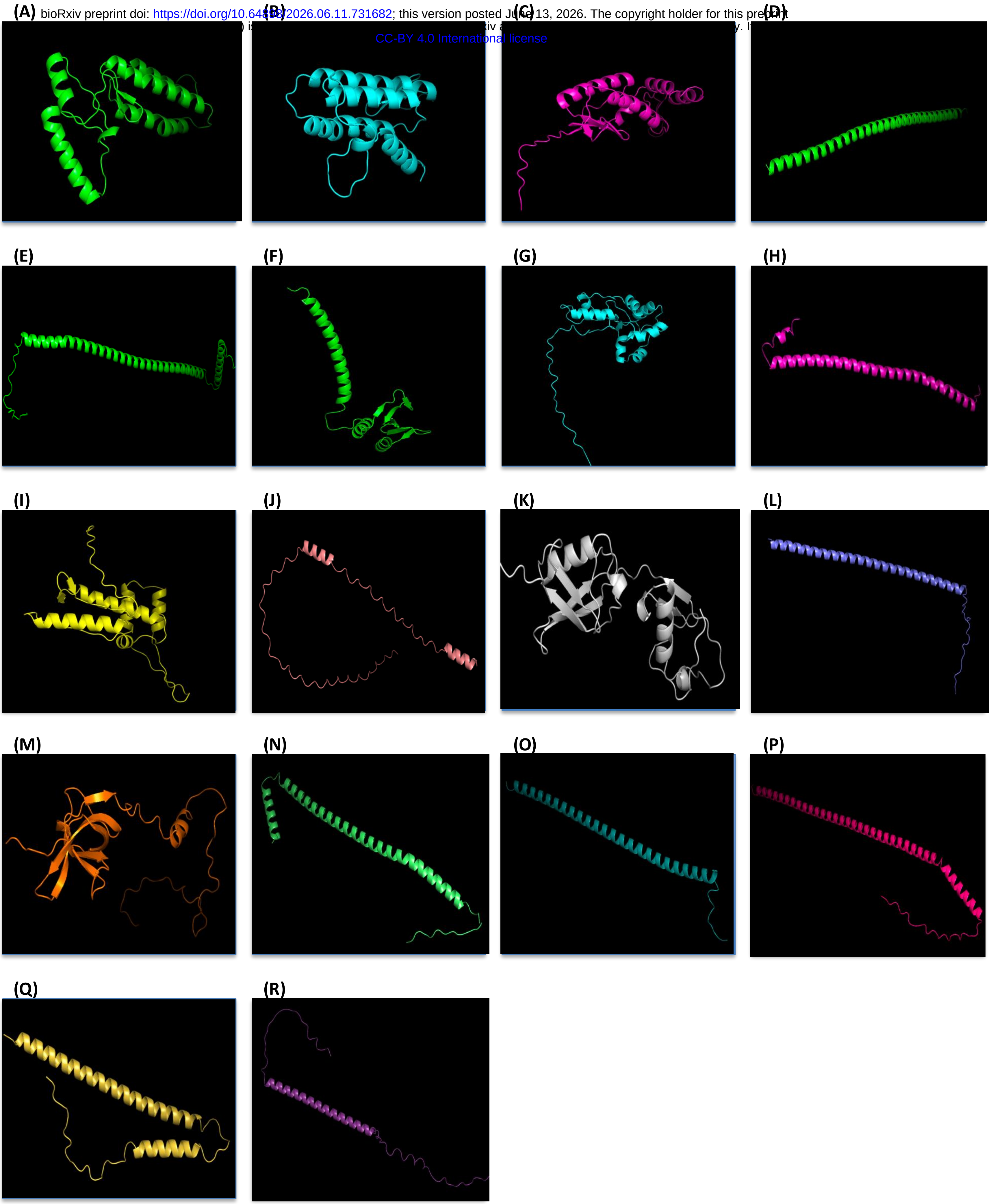

Supplement: Supplementary file 1 [file NIHPP2026.06.11.731682V1-supplement-1.pdf]
